# Supplementary figures and images for: The Servqual Method as an Assessment Tool of the Quality of Medical Services in Selected Asian Countries
Source: Int J Environ Res Public Health. 2022 Jun 26;19(13):7831. doi: 10.3390/ijerph19137831 (PMC9266116; doi:10.3390/ijerph19137831)

Suplementarny materials:

Tunnel plots for all dimensions:

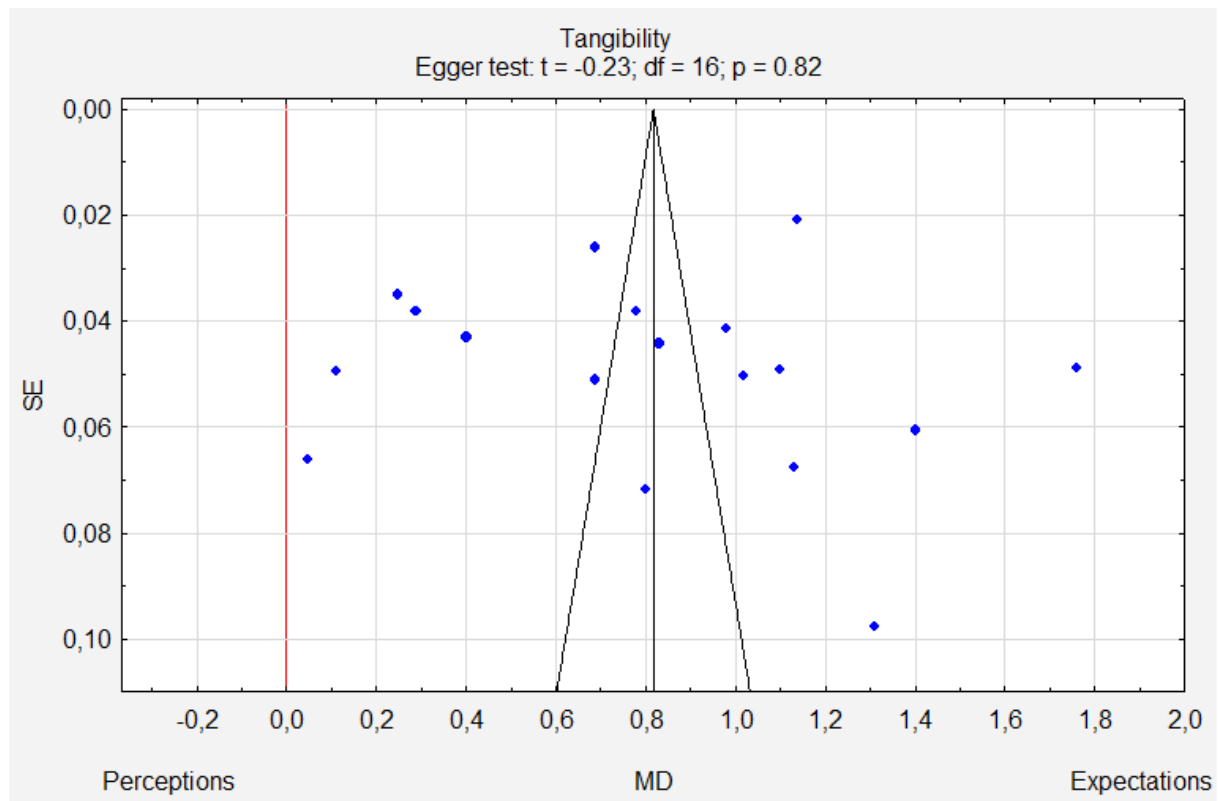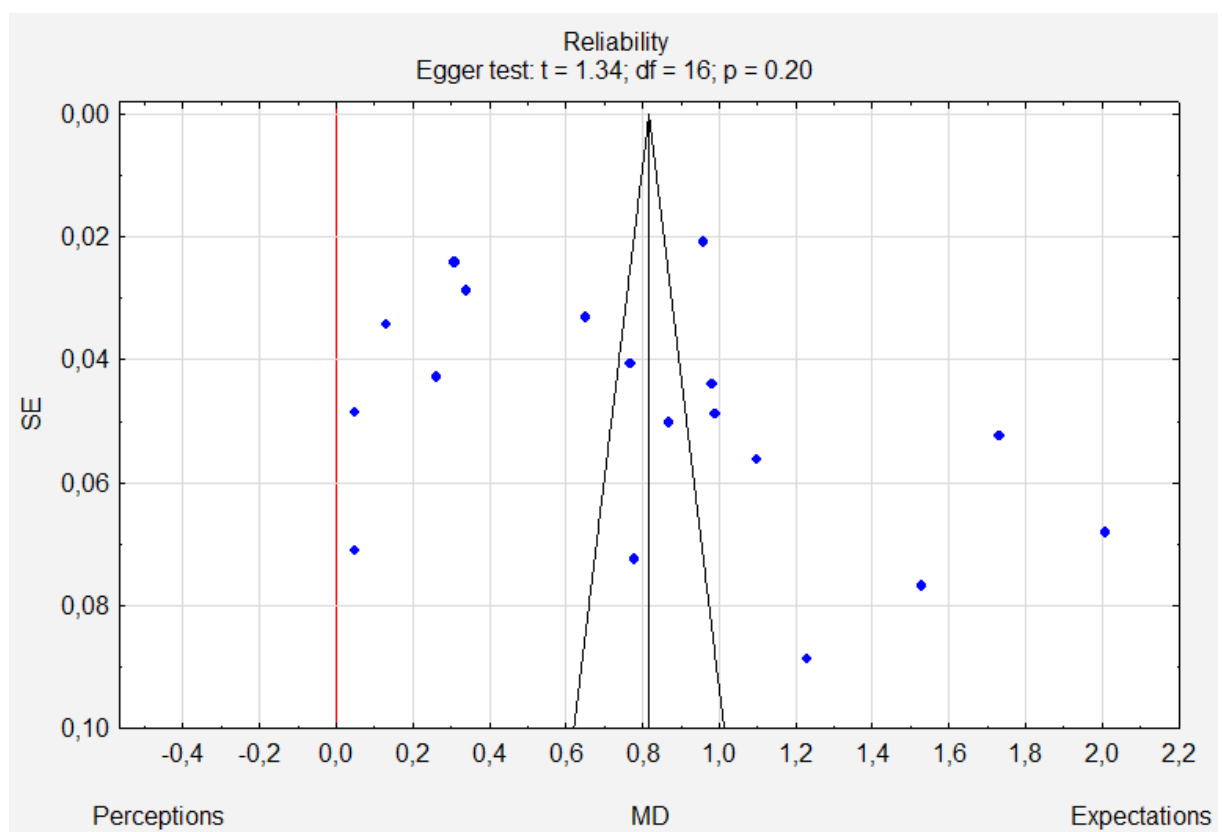

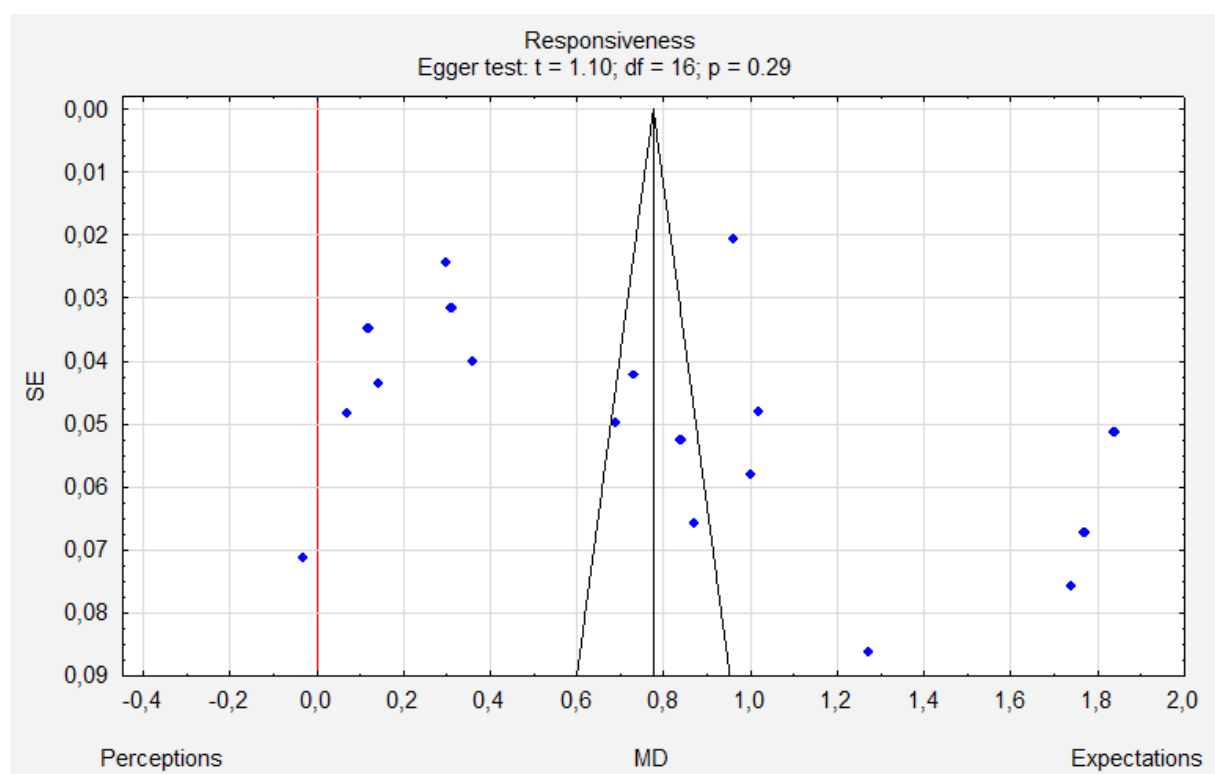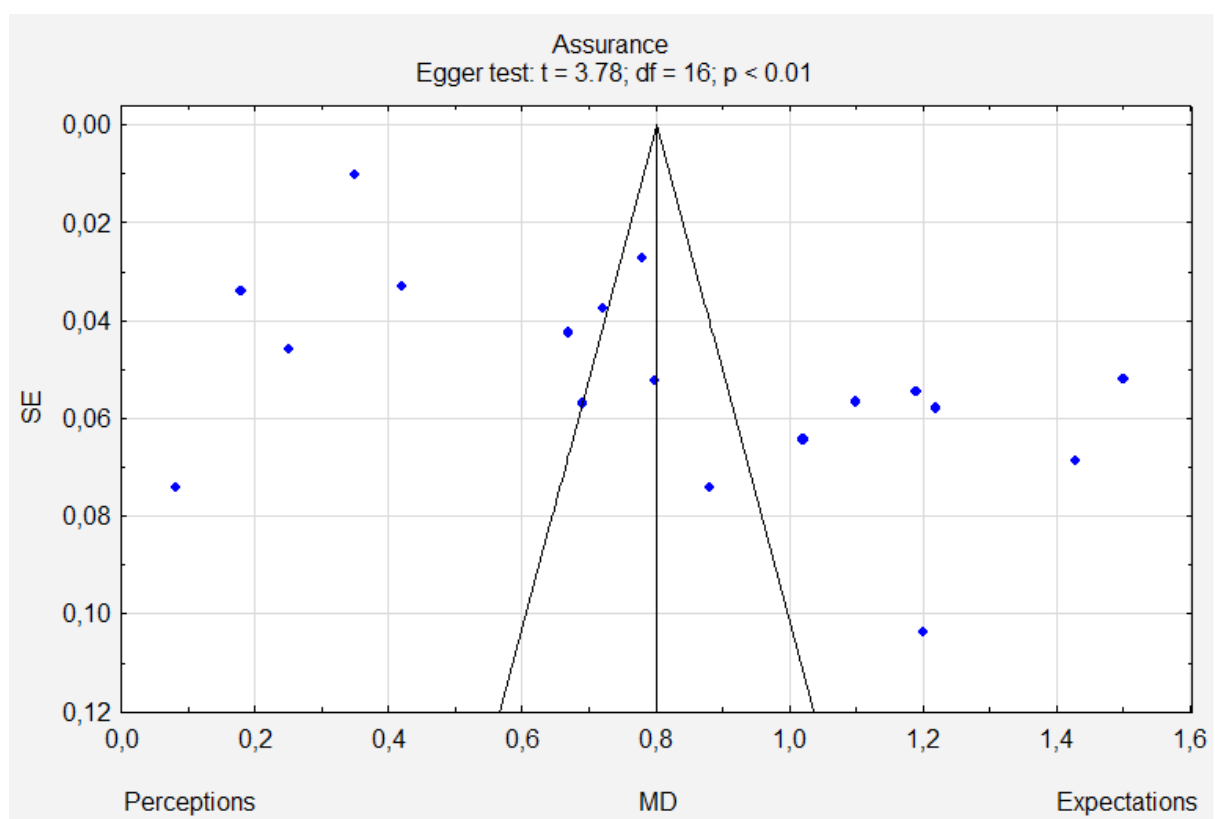

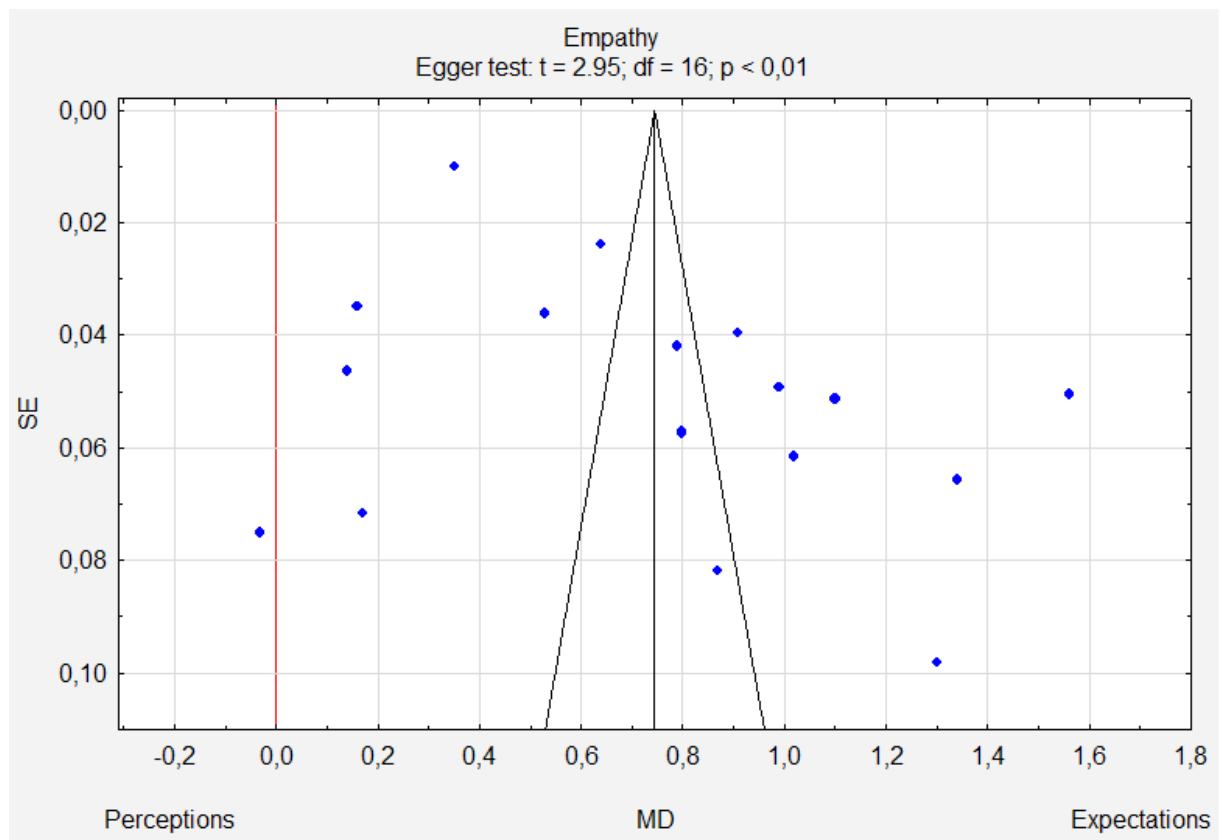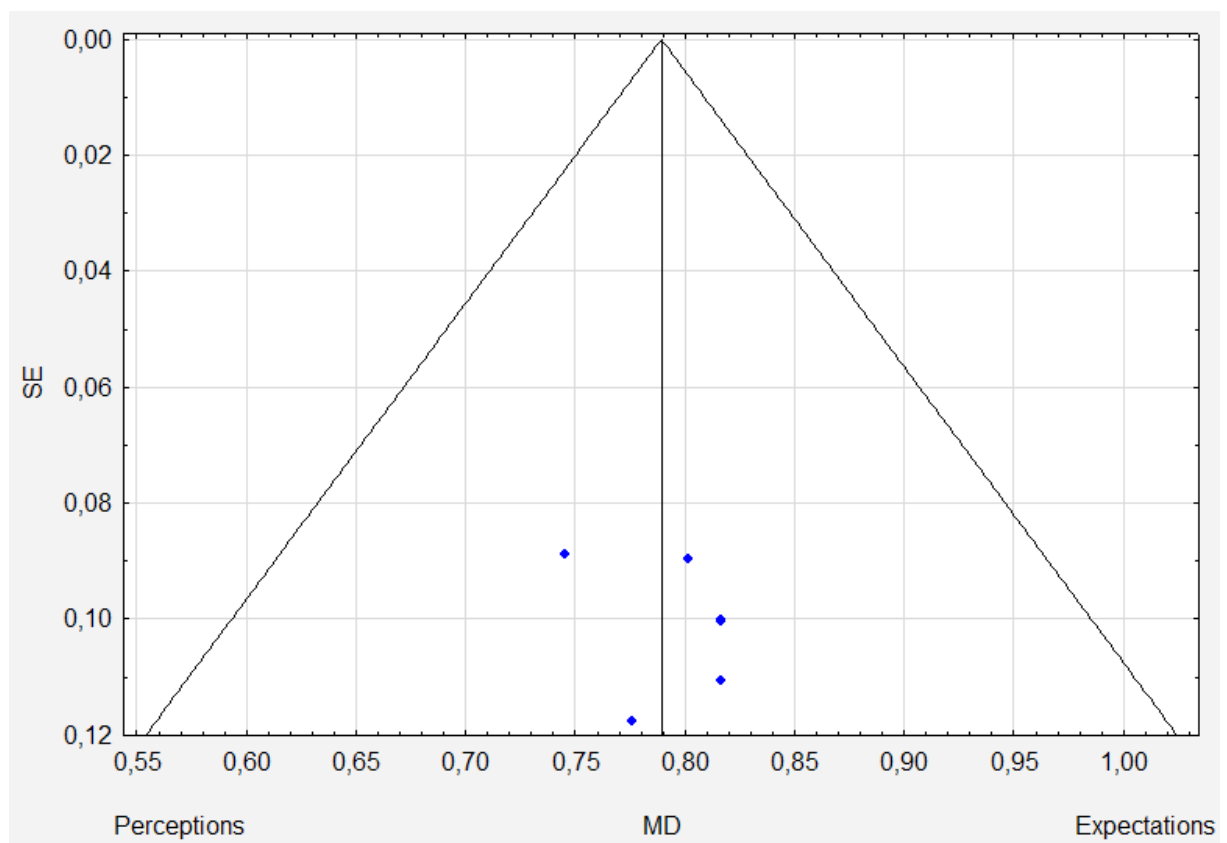

Supplement: Supplementary file 1 [file ijerph-19-07831-s001.zip › ijerph-1746619-supplementary.pdf]
